# Further Improvement of Debayering Performance of RGBW Color Filter Arrays Using Deep Learning and Pansharpening Techniques

**DOI:** 10.3390/jimaging5080068

**Published:** 2019-08-01

**Authors:** Chiman Kwan, Bryan Chou

**Affiliations:** Applied Research LLC., Rockville, MD 20850, USA

**Keywords:** debayering, RGBW pattern, CFA2.0, demosaicing, pansharpening, color filter array, Bayer pattern, deep learning

## Abstract

The RGBW color filter arrays (CFA), also known as CFA2.0, contains R, G, B, and white (W) pixels. It is a 4 × 4 pattern that has 8 white pixels, 4 green pixels, 2 red pixels, and 2 blue pixels. The pattern repeats itself over the whole image. In an earlier conference paper, we cast the demosaicing process for CFA2.0 as a pansharpening problem. That formulation is modular and allows us to insert different pansharpening algorithms for demosaicing. New algorithms in interpolation and demosaicing can also be used. In this paper, we propose a new enhancement of our earlier approach by integrating a deep learning-based algorithm into the framework. Extensive experiments using IMAX and Kodak images clearly demonstrated that the new approach improved the demosaicing performance even further.

## 1. Introduction

**Figure 1:**
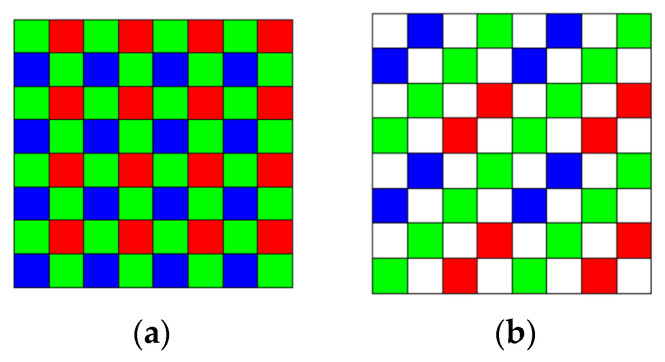
(**a**) Bayer pattern; **(b)** RGBW (aka color filter arrays (CFA2.0)) pattern.

Two mast cameras (Mastcams) are onboard the NASA’s rover, Curiosity. The Mastcams are multispectral imagers having nine bands in each. The standard Bayer pattern [1] in Figure 1a has been used for the RGB bands in the Mastcams. One objective of our research was to investigate whether or not it is worthwhile to adopt the 4 × 4 RGBW Bayer pattern [2,3] in Figure 1b instead of the 2 × 2 one in NASA’s Mastcams. We have addressed the comparison between 2 × 2 Bayer and 4 × 4 RGBW pattern in an earlier conference paper [4], which proposed a pansharpening approach. We observed that Bayer has better performance than the RGBW pattern. Another objective of our paper here is to investigate a new and enhanced pansharpening approach to demosaicing the RGBW images.

Compared to the vast number of debayering papers [2,5,6,7,8,9,10,11,12] for Bayer pattern [1], only few papers [2,3,4,13] talk about the demosaicing of the RGBW pattern. In [2,3], a spatial domain approach was described. In [13], a frequency domain approach was introduced. It was observed that the artifacts are less severe if RGBW is used in some demosaiced images. In [14], an improved algorithm known as least-squares luma–chroma demultiplexing (LSLCD) over [13] was proposed. Some universal algorithms [15,16,17] have been proposed in the last few years. In [18], optimal CFA patterns were designed for any percentage of panchromatic pixels. 

In a 2017 conference paper [4] written by us, a pansharpening approach was proposed to demosaicing the RGBW patterns. The idea was motivated by pansharpening [19,20,21,22,23,24,25,26,27,28,29,30,31,32,33,34], which is a mature and well-developed research area. The objective is to enhance a low resolution color image with help from a co-registered high resolution panchromatic image. Due to the fact that half of the pixels in the RGBW pattern are white, we think that it is appropriate to apply pansharpening techniques to perform the demosaicing. 

Although RGBW has some robustness against noise and low light conditions, it is not popular and does not have good performance [4] as compared to the standard Bayer pattern. Nevertheless, we would like to argue that the debayering of RGBW is a good research problem for academia even in the case where mosaiced images are clean and noise-free. Ideally, it will be good to reach the same level of performance of the standard Bayer pattern. However, it is a challenge to improve the debayering performance of RGBW.

In our earlier paper [4], our pansharpening approach consisted of the following steps. First, the generation of the pan band and the low resolution RGB bands is similar to that in [2,3]. Second, instead of downsampling the pan band, we apply some pansharpening algorithms to directly generate the pansharpened color images. However, the results in [4] were slightly better than the standard method [2,3] for IMAX data, but slightly inferior for Kodak data.

In this paper, we present a new approach that aims at further improving the pansharpening approach in [4]. There are two major differences between this paper and [4]. First, we propose to apply a recent deep learning based demosaicing algorithm in [35] to improve both the white band (also known as illuminance band or panchromatic band) and the reduced resolution RGB image. After that, a pansharpening step is used to generate the final demosaiced image. Second, it should be emphasized that a new “feedback” concept was introduced and evaluated. The idea is to feed the pansharpened images back to two early steps. Extensive experiments using the benchmark IMAX and Kodak images showed that the new framework improves over earlier approaches.

Our contributions are as follows:

- We are the first team to propose the combination of pansharpening and deep learning to demosaic RGBW pattern. Our approach opens a new direction in this research field and may stimulate more research in this area;
- Our new results improved over our earlier results in [4];
- Our results are comparable or better than state-of-the-art methods [2,14,16].

This paper is organized as follows. In Section 2, we will review the standard approach and also the pansharpening approach [4] of demosaicing the RGBW images. We will then introduce our new approach that combines deep learning and pansharpening. In Section 3, we will summarize our extensive comparative studies. Section 4 will include a few concluding remarks and future research directions.

## 2. Enhanced Pansharpening Approach to Demosaicing of RGBW CFAs

### 2.1. Standard Approach

**Figure 2:**
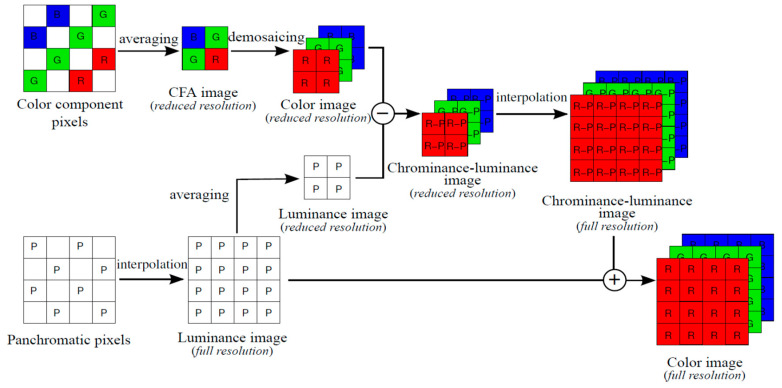
Standard approach to debayering CFA2.0 images. Image from [2].

In [3], a standard approach was presented. Figure 2 [2] depicts the key ideas. A mosaiced image is first split into color and panchromatic components. The color and the panchromatic components are then processed separately to generate the full resolution color images. This approach is very efficient and can achieve decent performance, which can be explained using Lemma 1 of [7]. For completeness, the lemma is included below.

**Lemma** **1.** *Let F be a full-resolution reference color component. Then any other full-resolution color components C ∈ {R, G, B} can be predicted from its subsampled version C_s_ using*

(1) $$C\approx I(C_{s}-F_{s})+F$$

*where F_s_ is subsampled version of F and I denotes a proper interpolation process.*

Lemma 1 provides a theoretical foundation for justifying the standard approach. Moreover, the standard approach is intuitive and simple.

### 2.2. Pansharpening Approach to Denosaicing CFA2.0 Patterns

**Figure 3:**
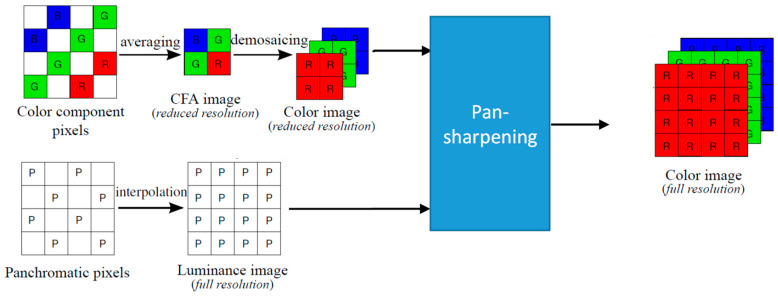
Pansharpening approach in [4].

Figure 3 shows our earlier pansharpening approach to debayering CFA2.0 images. Details can be found in [4]. The generation of pan and low resolution RGB images is the same in both Figure 2 and Figure 3.

In our earlier study [4], we applied nine representative pansharpening algorithms: Principal Component Analysis (PCA) [25], Smoothing Filter-based Intensity Modulation (SFIM) [27], Modulation Transfer Function Generalized Laplacian Pyramid (MTF-GLP) [28], MTF-GLP with High Pass Modulation (MTF-GLP-HPM) [29], Gram Schmidt (GS) [30], GS Adaptive (GSA) [31], Guided Filter PCA (GFPCA) [32], Partial Replacement based Adaptive Component-Substitution (PRACS) [33], and hybrid color mapping (HCM) [19,20,21,22,23].

In particular, HCM is a pansharpening algorithm that uses a high resolution color image to enhance a low resolution hyperspectral image. HCM can be used for color, multispectral, and hyperspectral images. More details about HCM can be found in [20] and open source codes can be found in [34].

### 2.3. Enhanced Pansharpening Approach 

**Figure 4:**
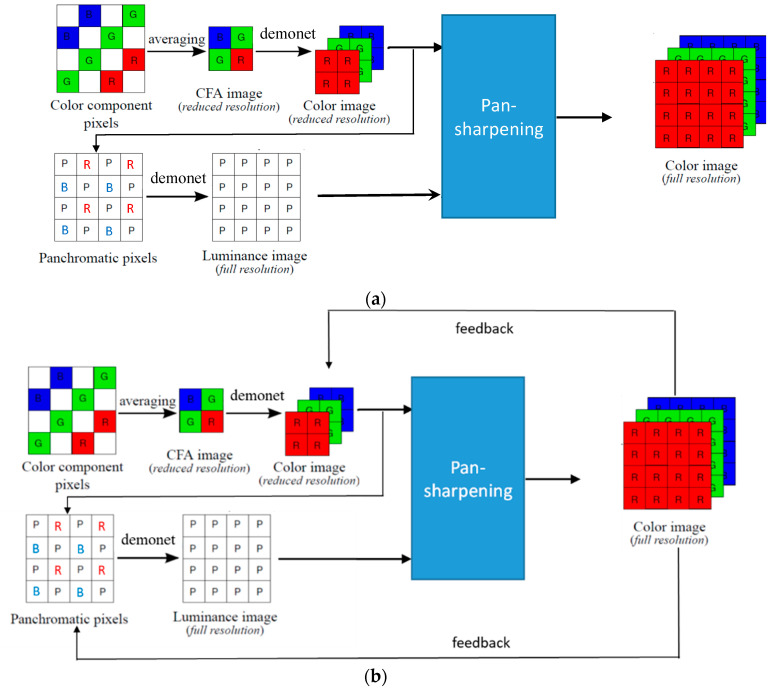
Proposed new deep learning based approach to demosaicing CFA2.0: (**a**) DEMONET based approach without feedback; (**b**) DEMONET based approach with feedback.

Figure 4 illustrates the enhanced pansharpening approach. First, we apply a deep learning based demosaicing algorithm known as DEMONET [35] to demosaic the reduced resolution CFA. Second, the demosaiced R and B images are upsampled and used to fill in the missing pixels in the panchromatic (pan) band. The reason for this is that the R and B bands have some correlations with the white pixels [36]. Some supporting arguments can be found below and also in Section 3.2. Third, we now treat the filled in pan band as a standard Bayer pattern with two white pixels, one R pixel, one B pixel, and then apply DEMONET again. The demosaiced image will have two white bands, one R band, and one B band. Fourth, the two white bands are averaged and extracted as the full resolution luminance band. Fifth, the luminance band is used to pansharpen the reduced resolution RGB images to generate the final demosaiced image. Sixth, we introduce a feedback concept (Figure 4b) that feeds the pansharpened RGB bands back to replace the reduced resolution RGB image and also replace those R and B pixels in the pan band. The pan band is then generated using DEMONET, and then pansharpening is performed again. This process repeats multiple times to yield the final results. We believe this “feedback” is probably the first ever idea in the demosaicing of RGBW images. Experimental results showed that the overall approach is promising and improved over earlier results in both IMAX and Kodak images. We observed that three iterations of feedback can generate good results.

**Figure 5:**
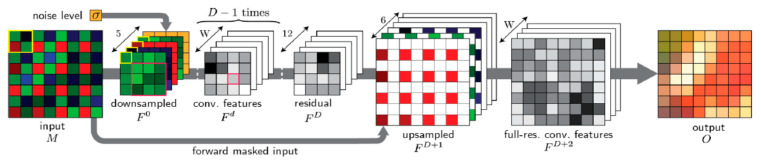
DEMONET architecture [35].

Here, we provide some more details about the DEMONET algorithm. We chose DEMONET because a comparative study was carried out in [35] that demonstrated its performance against other deep learning and conventional methods. As described in [35], the DEMONET is a feed-forward network architecture for demosaicing (Figure 5). The network comprises *D* + 1 convolutional layers. Each layer has W outputs and the kernel sizes are *K* × *K*. An initial model was trained using first network using 1.3 million images from Imagenet and 1 million images from MirFlickr. Additionally, some challenging images were searched to further enhance the training model. Details can be found in [35]. 

Some additional details regarding Figure 4 are described below.

**Figure 6:**
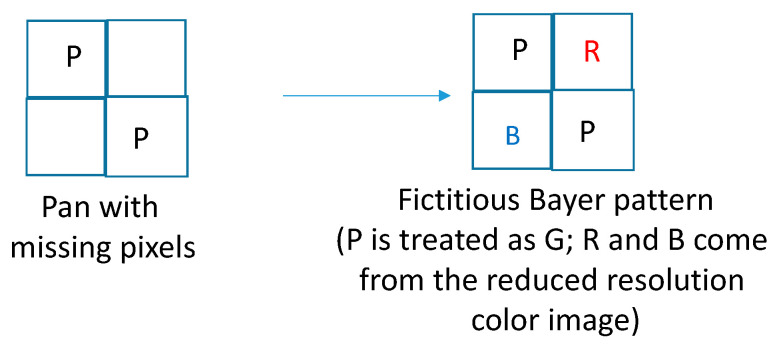
Fictitious Bayer pattern for pan band generation.

- First, we will explain how DEMONET was used for improving the pan band. Our idea was motivated by the research of [36] in which it was observed that the white (W) channel has a higher spectral correlation with the R and B channels than the G channel. Hence, we create a fictitious Bayer pattern where the original W (also known as P) pixels are treated as G pixels, the missing W pixels are filled in with interpolated R and B pixels from the low resolution RGB image. Figure 6 illustrates the creation of the fictitious Bayer pattern.

Once the fictitious Bayer pattern is created, we apply DEMONET to demosaic this pattern. The W or P pixels will be extracted from the G band in the demosaiced image. Although the above simple idea is very straightforward, the results of the improvement are quite large, which can be seen in Table 1.

**Figure 7:**
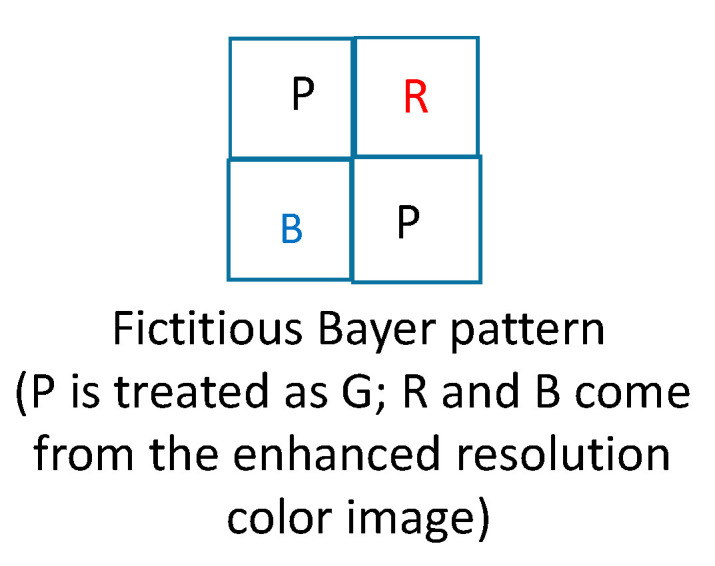
Fictitious Bayer pattern when there is feedback.

- Second, we would like to emphasize that we did not re-train the DEMONET because we do not have that many images. Most importantly, the DEMONET was trained with millions of diverse images. The performance of the above way of generating the pan band is quite good, as can be seen from Table 1;
- Third, we will explain how feedback works. There are two feedback paths. After the first iteration, we will obtain an enhanced color image. In the first feedback path, we replace the reduced resolution color image in Figure 4 with a downsized version of the enhanced color image. In the second feedback path, we directly replace the R and B pixels with the corresponding R and B pixels from the enhanced color image as shown in Figure 7.

We then apply DEMONET to the above enhanced Bayer pattern to generate an enhanced pan band and go through the pansharpening step to create another enhanced color image. The above process repeats three or more times. In our experiments, we found that the performance reaches the maximum after three iterations.

For ease of illustration of the work flow, we created a pseudo-code as follows:


**Combined Deep Learning and Pansharpening for Demosaicing RGBW Patterns**
Input: An RGBW patternOutput: A demosaiced color imageI = 1; iteration number
Step 1.For each 4 × 4 RGBW patch, create a 2 × 2 reduced resolution Bayer pattern, and also a 4 × 4 pan band with half of the pixels white and half of pixels missing. Repeat the above for the whole image.Step 2.Demosaic the 2 × 2 Bayer pattern using DEMONENT algorithm (pre-trained offline). Furthermore, upsample the demosaiced image to the same size of the original image.Step 3.Fill in the missing pixels of pan band.
Creation of a fictitious Bayer pattern for the pan band: Take R and B pixels from the upsampled demosaiced image and alternately fill in the missing pixels in the original pan band. Here, the green band of the fictitious Bayer pattern has pixels from the original white pixels in the pan band.Apply DEMONET to demosaic the fictitious Bayer pattern in Step 3a. Take the green band of the DEMONET output as the pan band.Replace half of the pixels in the output of Step 3b with the original white pixels in the original pan band.Step 4.Apply the HCM pansharpening algorithm to fuse the pan band from Step 3 and the reduced resolution color image from Step 2.
*     I = I + 1       If I > K, then stop. K is a pre-designed integer. We used K = 3 in our experiments.       Otherwise,
Step 5.Downsample the pansharpened image; feed it back to Step 2 to replace the reduced resolution color image.Step 6.Go to Step 3a, take R and B pixels from the pansharpened image, and fill them into those missing pixels in original pan band.Step 7.Repeat Steps 3b and 3c.Step 8.Repeat Step 4.
Go to *

One may ask why an end-to-end deep learning approach was not developed for RBGW. This is a good question for the research community and we do not have an answer for this at the moment. We believe that it is a non-trivial task to modify an existing scheme such as DEMONET to deal with RGBW. This extension by itself could be a good research direction for future research.

For the pansharpening module in Figure 4, we used HCM because it performed well in our earlier study [4].

## 3. Experimental Results

### 3.1. Data: IMAX and Kodak

**Figure 8:**
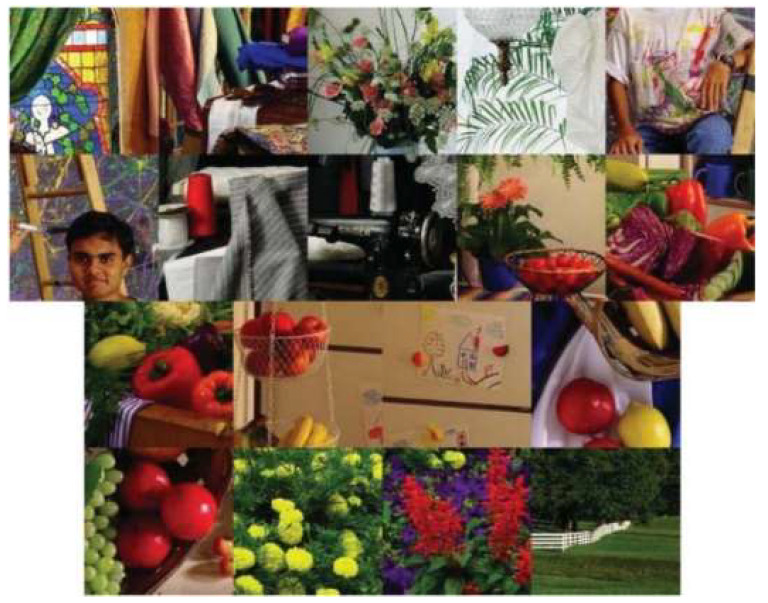
IMAX dataset (18 images).

**Figure 9:**
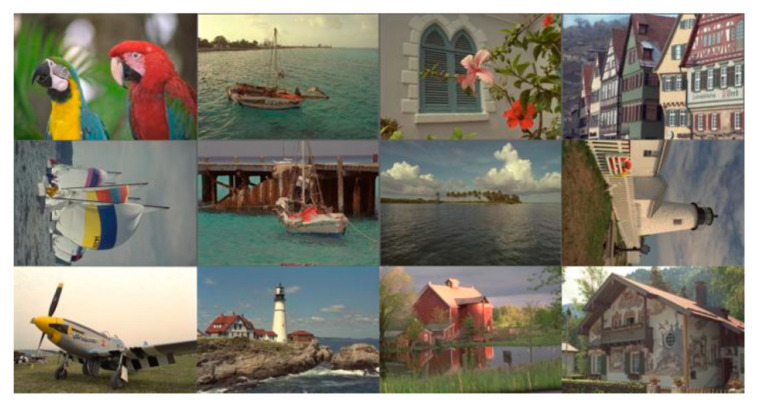
Kodak image set (12 images).

Similar to earlier studies in the literature, we used IMAX (Figure 8) and Kodak (Figure 9) data sets. In the original Kodak data, there are 24 images. We chose only 12 images because other researchers [2] also used these 12 images.

### 3.2. Performance Metrics and Comparison of Different Approaches to Generating the Pan Band

Two well-known performance metrics were used: Peak signal-to-noise ratio (PSNR) and CIELAB [37]. In Table 1, we first show some results that justify why we fill in the R and B pixels in the missing locations of the panchromatic band. Table 1 shows the PSNR values of several methods for generating the pan band. It can be seen that the bilinear and Malvar-He-Cutler (MHC) methods have 31.26 and 31.91 dBs, respectively. To explore alternatives for generating better pan band, we used DEMONET with filled in R and B pixels from two cases (one from the reduced resolution color image and one from the ground truth RGB images). We can clearly see that the PSNR values (33.13 and 37.48) are larger with DEMONET than those by using bilinear and MHC methods. This is because the R and B pixels have some correlations with the white pixels and DEMONET was able to extract some information from the R and B pixels in the demosaicing process. In practice, we will not have the ground truth RGB bands and hence the 37.4825 dBs will never be attained. However, as shown in Figure 4b, we can still take R and B values from the pansharpened RGB image. It turns out that such a feedback process further enhances the performance of our proposed method. We believe the above “feedback” idea is a good contribution to the demosaicing community for CFA2.0.

In our study, we also did customize the deep learning demosaicing method for Mastcam images from NASA because Mastcam images are of interest to NASA. It is interesting to observe that our customized model did not perform as well as the original model. This is because (1) our Mastcam image database is limited in size; (2) the original DEMONET used millions of images. Based on the above, we decided to use the original model instead of re-training it. In other words, if the original model is already good enough, there is no need to re-invent the wheel.

### 3.3. Evaluation Using IMAX Images

Table 2 summarizes the PSNR and CIELAB scores for the IMAX images. The column “Before Processing” contains results using the bicubic interpolation of the reduced resolution color image in Figure 4. We could have included results using some other RGBW demosaicing algorithms [13,14,15,16,17]. However, we contacted those authors for their codes. Some [13,15] did not respond and some [16,17] provided codes that were not for the RGBW pattern. Actually, we tried to implement some of those algorithms [16,17], but could not get good results. We were able to obtain LSLCD codes from [14] and have included comparisons with [14] in this paper. The column “Standard” refers to results using the standard demosaicing procedure in Figure 2. The column “LSLCD” shows results using the algorithm from [14]. The “HCM” contains results using the framework in Figure 3. The last two columns contain the results generated by using the proposed new framework (without and with feedback) in Figure 4. It can be seen that the new framework with feedback based on DEMONET achieved better results in almost all images as compared to the earlier approaches. The improvement is about 0.8 dBs over the best previous approach in terms of averaged PSNR for all images.

**Figure 10:**
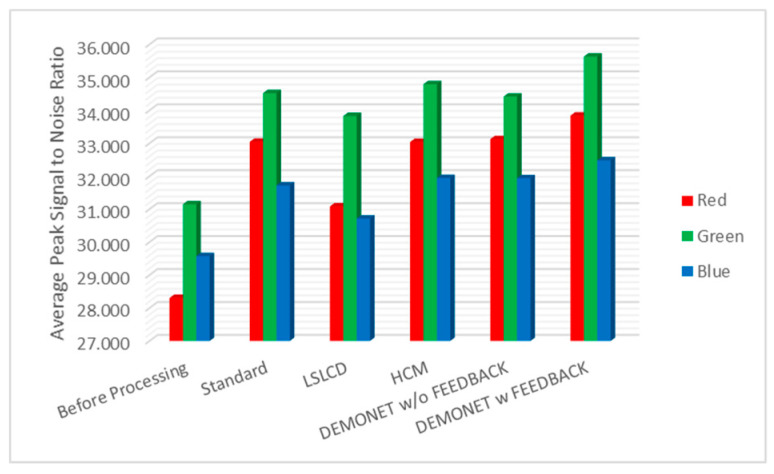
Averaged PSNR values of different methods for RGB bands.

**Figure 11:**
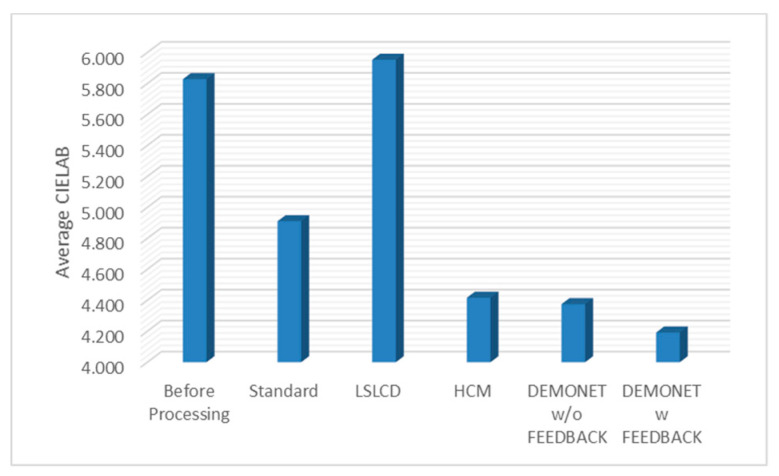
Averaged CIELAB values of different methods.

**Figure 12:**
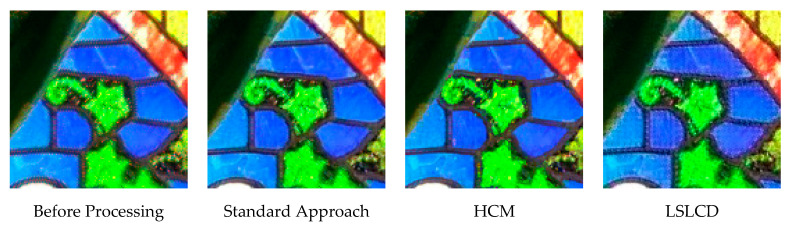

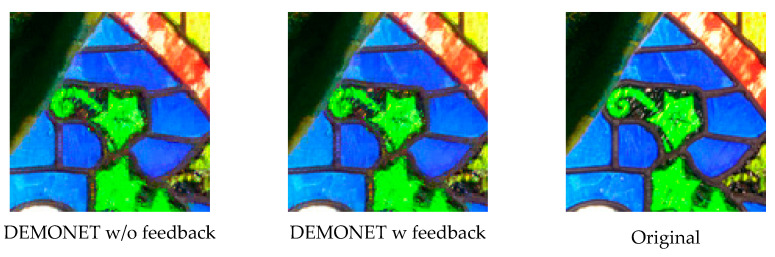
Demosaiced images of different algorithms for one IMAX image.

Figure 10 and Figure 11 depict the averaged PSNR and CIELAB scores of the various methods for IMAX images. The scores of the new framework are better than earlier methods. Figure 12 visualizes all the demosaiced images as well as the original image for one IMAX image. It can be seen that the images using the new framework are comparable to others.

### 3.4. Evaluation Using Kodak Images

**Figure 13:**
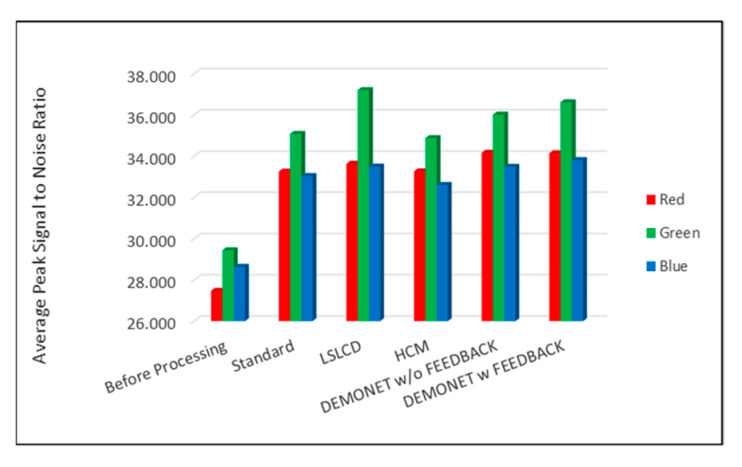
Averaged PSNR values of different methods for RGB bands.

**Figure 14:**
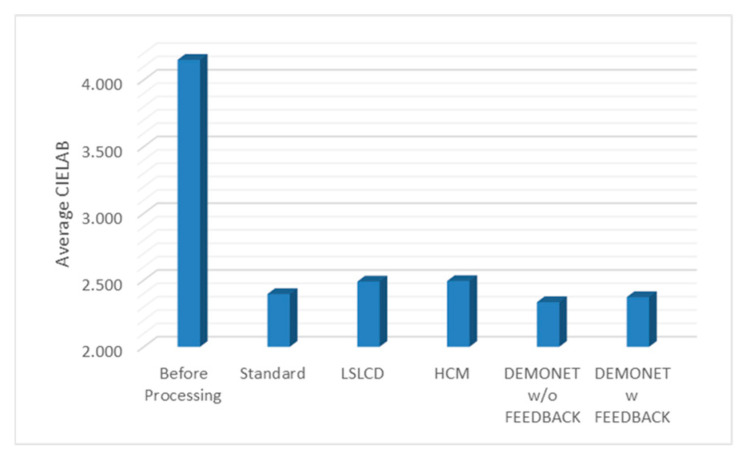
Averaged CIELAB values of different methods.

**Figure 15:**
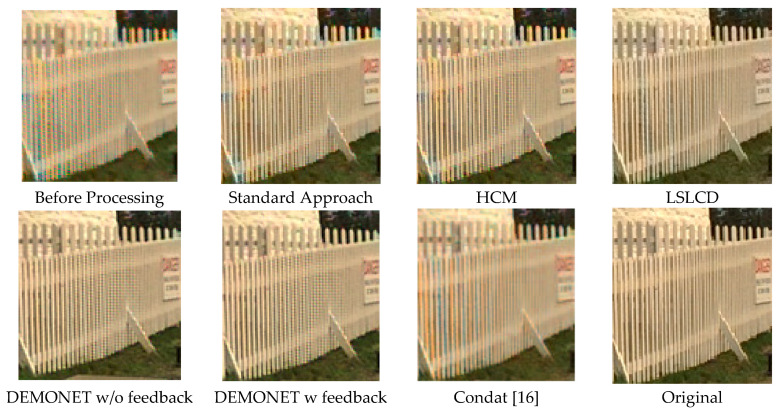
Demosaiced Kodak images using various algorithms.

Table 3 summarizes the PSNR and CIELAB scores of various algorithms for the Kodak images. The arrangement of columns in Table 3 is similar to that in Table 2. We observe that the new approach based on DEMONET yielded better results than most of the earlier methods. Figure 13 and Figure 14 plot the averaged PSNR and CIELAB scores versus different algorithms. The averaged CIELAB scores of the proposed approach without and with feedback are close to each other to the third decimal place. In terms of PSNR, the approach with feedback is 0.3 dBs better than that without feedback. In general, Kodak images have better correlations between bands than that of IMAX images according to [5]. Because of the above observation, algorithms working well for Kodak images may not work well for IMAX images. Figure 15 shows the demosaiced images of various algorithms. We also included one demosaiced image from one universal demosaicing algorithm [16] in Figure 15. We can see that results using proposed framework with DEMONET look slightly better than the other methods in terms of color distortion. 

## 4. Conclusions

We present a deep learning-based approach that improves an earlier pansharpening approach to debayering CFA2.0 CFAs. Our key idea is to utilize the deep learning-based algorithm to improve the interpolation of the illuminance/pan band and also the reduced resolution color image. A novel feedback concept was introduced that can further enhance the overall demosaicing performance. Using IMAX and Kodak data sets, we carried out a comparative study between the proposed approach and earlier approaches. One can observe that the proposed new approach has better performance than earlier approaches for both the Kodak data and the IMAX data. 

One future research direction is on how to improve the quality of the pan band. Another direction is to develop a stand-alone and end-to-end deep learning based approach for RGBW patterns.

## Figures and Tables

**Table 1:** Peak signal-to-noise ratio (PSNR) of pan bands generated by using different interpolation methods.

Interpolation Method	PSNR
BILINEAR	31.2574
Malvar-He-Cutler (MHC)	31.9133
DEMONET using R and B pixels from the Reduced resolution RGB	33.1281
DEMONET using R and B pixels from the GROUND TRUTH RGB image	37.4825

**Table 2:** PSNR and CIELAB metrics of different algorithms: IMAX data. Bold numbers indicate the best performing method in each row.

Image	Metric	Before Processing	Standard	LSLCD	HCM	DEMONET w/o FEEDBACK	DEMONET w FEEDBACK
1	PSNR	23.965	26.203	24.648	**26.210**	25.783	26.182
	CIELAB	9.447	8.182	9.611	7.755	7.897	**7.682**
2	PSNR	28.879	**32.812**	31.594	32.354	32.010	32.759
	CIELAB	6.384	5.459	6.524	5.105	5.053	**4.858**
3	PSNR	24.390	28.917	29.563	28.880	29.026	**30.085**
	CIELAB	8.777	6.400	6.833	5.806	6.028	**5.635**
4	PSNR	26.838	31.528	31.699	32.980	32.386	**34.061**
	CIELAB	3.887	2.723	2.935	2.101	2.286	**2.080**
5	PSNR	27.709	30.433	28.937	31.460	30.968	**31.503**
	CIELAB	5.087	4.107	5.073	**3.753**	4.073	3.901
6	PSNR	31.355	32.866	30.795	33.715	33.538	**33.999**
	CIELAB	4.530	3.995	5.014	3.600	3.705	**3.583**
7	PSNR	28.637	33.545	34.391	32.293	33.547	**34.786**
	CIELAB	5.045	3.340	3.484	3.654	2.991	**2.836**
8	PSNR	29.614	34.583	34.438	34.239	34.610	**36.118**
	CIELAB	6.441	5.235	6.394	5.198	5.101	**4.884**
9	PSNR	30.964	34.298	32.133	**34.899**	33.962	34.869
	CIELAB	4.867	4.325	5.561	3.584	3.474	**3.304**
10	PSNR	32.068	35.430	33.407	34.996	34.982	**35.518**
	CIELAB	5.191	4.458	5.754	4.076	4.198	**4.038**
11	PSNR	33.733	36.475	34.332	36.364	36.654	**37.203**
	CIELAB	5.523	5.159	6.704	4.206	4.043	**3.893**
12	PSNR	29.501	34.629	34.493	34.982	35.126	**36.358**
	CIELAB	4.289	3.091	3.700	2.886	2.908	**2.697**
13	PSNR	34.374	38.003	36.480	38.686	38.542	**39.245**
	CIELAB	2.285	1.825	2.186	1.729	1.760	**1.705**
14	PSNR	33.535	36.651	35.852	36.826	36.545	**37.080**
	CIELAB	3.784	3.357	3.919	3.175	3.224	**3.147**
15	PSNR	34.716	37.254	35.470	37.610	37.010	**37.641**
	CIELAB	4.208	3.992	5.306	**3.586**	3.805	3.677
16	PSNR	27.638	29.756	28.101	31.011	30.811	**31.567**
	CIELAB	9.417	8.560	9.837	6.578	6.053	**5.793**
17	PSNR	28.159	**29.222**	26.304	28.806	28.528	28.927
	CIELAB	9.477	9.381	12.852	8.330	8.329	**8.111**
18	PSNR	28.113	33.122	31.152	32.376	32.857	**33.840**
	CIELAB	6.270	4.808	5.475	4.368	3.828	**3.636**
Average	PSNR	29.677	33.096	31.877	33.260	33.160	**33.986**
	CIELAB	5.828	4.911	5.954	4.416	4.375	**4.192**

**Table 3:** PSNR and CIELAB metrics of various algorithms: Kodak data. Bold numbers indicate the best performing method in each row.

Image	Metric	Before Processing	Standard	LSLCD	HCM	DEMONET w/o FEEDBACK	DEMONET w FEEDBACK
1	PSNR	33.018	37.560	32.111	37.095	**37.994**	37.986
	CIELAB	2.123	**1.534**	3.646	1.632	1.623	1.657
2	PSNR	26.305	31.862	**36.086**	31.756	33.306	33.827
	CIELAB	4.869	2.719	**2.022**	2.820	2.485	2.518
3	PSNR	31.690	**36.777**	34.484	36.180	36.433	36.616
	CIELAB	2.936	**1.877**	2.831	2.103	2.155	2.198
4	PSNR	22.690	29.447	31.288	29.536	30.780	**31.439**
	CIELAB	7.593	3.608	3.343	3.608	3.211	**3.196**
5	PSNR	30.919	36.883	35.855	36.742	37.534	**37.884**
	CIELAB	2.469	1.424	1.783	1.452	**1.400**	1.430
6	PSNR	27.652	32.932	**35.045**	32.613	33.615	33.866
	CIELAB	5.110	2.918	**2.885**	3.139	3.110	3.206
7	PSNR	29.738	35.484	**39.361**	35.385	36.821	37.307
	CIELAB	3.813	2.123	**1.519**	2.164	1.881	1.907
8	PSNR	26.933	33.454	**35.077**	33.394	34.466	34.816
	CIELAB	4.562	2.538	**2.116**	2.722	2.501	2.564
9	PSNR	30.288	35.407	36.015	35.186	35.826	**36.229**
	CIELAB	2.871	**1.766**	1.954	1.867	1.777	1.813
10	PSNR	27.065	32.453	**34.956**	32.315	33.514	33.756
	CIELAB	4.572	2.698	**2.413**	2.758	2.529	2.606
11	PSNR	28.571	33.534	33.472	33.207	33.790	**33.804**
	CIELAB	4.115	**2.679**	2.979	2.750	2.726	2.756
12	PSNR	25.367	29.691	**33.538**	29.561	30.478	30.702
	CIELAB	4.766	2.860	**2.375**	2.905	2.620	2.616
Average	PSNR	28.353	33.790	34.774	33.581	34.546	**34.853**
	CIELAB	4.150	2.395	2.489	2.493	**2.335**	2.372
